# Role of Sodium Nitroprusside on Potential Mitigation of Salt Stress in Centaury (*Centaurium erythraea* Rafn) Shoots Grown In Vitro

**DOI:** 10.3390/life13010154

**Published:** 2023-01-05

**Authors:** Milana Trifunović-Momčilov, Nikola Stamenković, Marija Đurić, Snežana Milošević, Marija Marković, Zlatko Giba, Angelina Subotić

**Affiliations:** 1 Department for Plant Physiology, Institute for Biological Research “Siniša Stanković”—National Institute of Republic of Serbia, University of Belgrade, Bulevar despota Stefana 142, 11060 Belgrade, Serbia; 2 Faculty of Biology, University of Belgrade, Studentski trg 16, 11000 Belgrade, Serbia

**Keywords:** common centaury, salinity stress, oxidative stress, antioxidative protection, sodium nitroprusside

## Abstract

Soil salinity is one of the most common abiotic stressors that affects plant growth and development. The aim of this work was to investigate the influence of sodium nitroprusside (SNP), a donor of nitric oxide (NO), on the physiological response of common centaury (*Centaurium erythraea*) shoots grown under stress conditions caused by sodium chloride (NaCl) in vitro. Centaury shoots were first grown on nutrient medium containing different SNP concentrations (50, 100 and 250 μM) during the pretreatment phase. After three weeks, the shoots were transferred to nutrient media supplemented with NaCl (150 mM) and/or SNP (50, 100 or 250 μM) for one week. The results showed that salinity decreased photosynthetic pigments, total phenolic content and DPPH (1,1-diphenyl-2-picrylhydrazyl radical) concentration. The activities of antioxidant enzymes, namely superoxide dismutase (SOD), catalase (CAT) and peroxidase (POX), were also reduced under salt stress. However, MDA concentration was decreased, while H_2_O_2_ and proline content did not drastically change under the stress conditions caused by NaCl. Exogenous application of SNP altered the biochemical parameters of centaury shoots grown under salt stress. In this case, increased photosynthetic pigment content, total phenolics and proline content were noted, with reduced MDA, but not H_2_O_2_, concentration was observed. In addition, the exogenous application of SNP increased the degree of DPPH reduction as well as SOD, CAT and POX activities.

## 1. Introduction

Environmental conditions are rarely ideal and plants are constantly exposed to various types of stress during their life cycle. Stress can be defined as a factor that decreases the rate of physiological processes that negatively affects growth, development and plant productivity [1]. In the context of energy consumption, stress can be observed as a state in which reduced energy production is directed towards stress-defense processes rather than growth and development [2]. In natural conditions, plants are mainly exposed to a combination of different stress-inducing factors that interact with each other and modify their individual effects accordingly. Salt stress is one of the major abiotic factors limiting crop productivity. According to Shrivastava and Kumar [3], more than 50% of lands are affected by salinity, while salinized areas have a tendency to increase by 10% every year. Since almost all food originates from soil, it is more than clear what problem salinization presents to the food supply [4]. In addition to natural salinization, which is the accumulation of dissolved salts in the soil to the levels that interfere with agricultural production and environment, there is also secondary salinization that occurs as a result of anthropogenic influences [5].

Salt stress disrupts plant homeostasis in two ways. First, high concentrations of salt in the soil prevent water uptake by the roots, while the accumulation of salts in plants, primarily Na^+^ and Cl^-^ ions, further leads to toxic effects [6]. During the initial phase of defense against salt stress, water deficit and osmotic stress causes a decrease in cell division rate in leaves, root and shoot meristems [7]. Osmotic stress also leads to stomatal closure and reduction in photosynthesis efficiency [8]. The next phase of the plant’s defense against salt stress occurs due to accumulation of toxic ions, leading to damage of cell membranes’ structure and function, inhibition of enzyme activity and finally, plant productivity [9]. Secondary oxidative stress follows immediately after primary stressors, osmotic stress and ion toxicity. Oxidative stress is a complex chemical and physiological phenomenon that occurs as a result of intensive production and accumulation of reactive oxygen species (ROS) which, due to high reactivity, damage proteins, lipids and nucleic acids [10]. By damaging the lipids, the integrity and functions of membranes deteriorate. These fragmentation products can further damage proteins and nucleic acids, thereby interfering with the normal functioning of receptors, enzymes and membrane channels, resulting in cell death [11]. Accordingly, lipid oxidation, also known as lipid peroxidation, is one of the markers of oxidative stress.

Since prolonged exposure to stress leads to cell death, plants have developed numerous mechanisms that enable growth in different stress conditions. Tolerance to salt stress is a complex phenomenon involving numerous regulatory processes such as stomatal opening, changes in hormonal balance, activation of antioxidant defense systems, osmotic adjustment, maintenance of water balance, export of toxic ions or their compartmentalization in vacuoles [12]. Antioxidant defense mechanisms are divided into two groups, non-enzymatic and enzymatic. Both groups of antioxidants are involved in protecting cellular components from oxidation as well as conversion of ROS into less reactive forms. In addition to ascorbic acid, glutathione, tocopherols, polyamines and phenols, proline and glycine betaine are the most important non-enzymatic components [9]. Proline is one of the essential amino acids, with great importance in protein synthesis. The accumulation of proline in plant cell results after different disturbances to the external environment [13]. In addition, proline is known to regulate the expression of genes important for mitochondrial stability, cell division and cell death [9,14,15]. Several different enzymes such as superoxide dismutase (SOD), catalase (CAT), peroxidase (POX), glutathione peroxidase (GPX), glutathione reductase (GR), glutathione S-transferases (GST), ascorbate peroxidase (APX), monodehydroascorbate reductase (MDHAR), and dehydroascorbate reductase (DHAR), act as part of the plant antioxidant defense system [16]. In addition, SOD is considered one of the major enzymatic systems that scavenges stress-generated free radicals in plants while other enzymes such as CAT and POX, work in close synchrony with SOD to prevent the formation of more harmful ROS through the Haber–Weiss reaction.

Nitric oxide (NO) is a paramagnetic molecule with an unpaired π∗ electron, which can easily diffuse through membranes [17]. Initially, NO was considered an air pollutant that inhibits plant growth and denatures DNA, damages lipids, and decreases intensity of photosynthesis and respiration [18]. Today, however, it is known that NO is an important molecule in redox signaling, participates in the control of numerous physiological processes and plays an important role in establishing resistance to pathogens and regulating the plant’s response to abiotic stress [19,20,21]. Sodium nitroprusside (SNP), a common NO donor, plays diverse roles in plant growth and development. Numerous studies have confirmed the protective role of SNP during salt stress conditions in tomato [22], cucumber [23], orange [24], cotton [25], alfalfa [26], apple [27], wheat [28] and lentil [29] plants.

Centaury (*Centaurium erythraea* Rafn) is medicinal plant that is widely used in traditional medicine as an antidiabetic, antipyretic, antiflatulent and detoxifying agent [30]. Various bioactive compounds isolated from the aerial part of centaury have shown different therapeutic properties [30,31,32,33,34,35,36]. Among the species belonging to the *Centaurium* genus, centaury is the plant species to which the greatest attention has been paid during recent years. The first and most important reason is the relatively easy manipulation of this plant species, which makes it an excellent model system for studying genetic transformation, secondary metabolites and salt stress physiology [37,38,39,40]. Moreover, centaury has recently shown to possess great developmental plasticity and the ability to induce somatic embryogenesis in root and leaf cultures [41]. A previous report described the salinity-stress response of centaury shoots and roots grown in vitro [38]. In this work, we investigated whether exogenous application of SNP can alleviate the effects of stress caused by NaCl in centaury shoots grown in vitro.

## 2. Materials and Methods

### 2.1. Plant Material, Culture Conditions and Experimental Design

**Figure 1:**
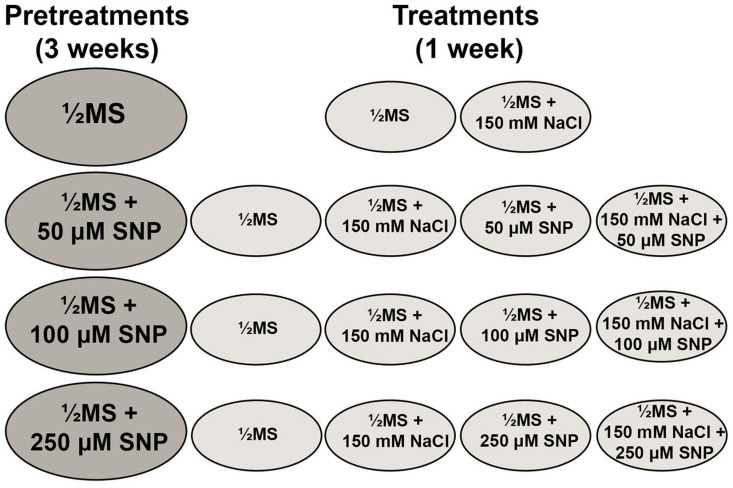
Schematic of experimental design including different SNP pretreatments and NaCl and/or SNP treatments.

Mother stock cultures of centaury plants were used as the primary plant material. The centaury shoots were cultured in vitro, on half-strength MS medium (½MS, [42]) solidified with 0.7% agar and supplemented with 3% sucrose as well as 100 mg L^−1^ *myo*-inositol. The medium was adjusted to pH 5.8 with NaOH/HCl and autoclaved at 121 °C for 25 min. All in vitro cultures were grown at 25 ± 2 °C and a 16/8 h light/dark photoperiod (“Tesla” white fluorescent lamps, 65 W, 4500 K; light flux of 47 μmol s^−1^ m^−2^). During the three-week long pretreatment, the centaury shoots were first placed on four types of ½MS nutrient media containing different SNP concentrations (0, 50, 100 or 250 μM). After pretreatment, the centaury shoots were transferred to fresh ½MS nutrient media supplemented with NaCl (0 or 150 mM) and/or SNP (0, 50, 100 or 250 μM) and cultured for one week (Figure 1). All experiments were repeated three times.

### 2.2. Quantification of Photosynthetic Pigments

Isolation of total chlorophyll (*Chl*) and carotenoids was accomplished from the leaves collected from the bottom part of the centaury rosette after four weeks of cultivation. Total *Chl* and carotenoid content were extracted using 96% ethanol as proposed by Lichtenthaler [43] and previously described in detail by Trifunović-Momčilov et al. [39]. The absorbance of the photosynthetic pigments was measured using a UV–visible spectrophotometer (Agilent 8453, Life Sciences, Santa Clara, CA, USA).

### 2.3. Estimation of Oxidative Stress Biomarkers

The level of lipid peroxidation was measured as malondialdehyde (MDA) concentration by the procedure described by Heath and Packer [44], while H_2_O_2_ concentration was determined as described by Velikova et al. [45]. In both assays, 0.1% trichloroacetic acid was used and detailed protocols were previously described by Trifunović-Momčilov et al. [38]. The spectrophotometric determination of MDA and H_2_O_2_ were measured using an ELISA Micro Plate Reader (LKB 5060–006, Winooski, VT, USA).

### 2.4. Estimation of Nonenzymatic Antioxidants

Free proline content was determined by the ninhydrin reaction which consists of the reaction of proline and ninhydrin reagent (2,2-dihydroxyindane-1,3-dione) resulting in a yellow reaction product [46]. Proline extraction and measurement was performed according to a modified method by Carillo and Gibon [47] and described in detail by Trifunović-Momčilov et al. [38].

Total polyphenol content was determined using the Folin–Ciocalteu test (FC test) based on reaction of polyphenols from plant tissues and Folin–Ciocalteu reagents forming a blue-colored complex that can be spectrophotometrically quantified. This method was previously described by Singleton et al. [48]. The plant material (200 mg) was homogenized in liquid nitrogen and extracted with 96% ethanol. The homogenate was incubated for 60 min at room temperature and then centrifuged for 15 min. The supernatant was further mixed with the FC reagent solution, which was previously prepared by adding distilled water to the FC reagents in a volume ratio of 2:1. The reaction mixture was quickly vortexed and 20% Na_2_CO_3_ was added. After 90 min at room temperature in darkness, the absorbance was measured at 765 nm. In this assay, gallic acid was used as a phenol standard.

The antioxidant activity in the centaury shoots was determined after evaluation of stable DPPH radical concentrations. The samples were prepared using the same method as the FC test. In the reaction with antioxidants, the DPPH radical is converted to a non-radical form through reduction by hydrogen ions. After homogenization and centrifugation of the supernatant, methanol and DPPH reagent solution were added. The reaction mixture was incubated at room temperature in the dark. After 60 min, the degree of reduction of the DPPH radical was estimated through an absorbance measurement at 520 nm. The scavenging capacity of the DPPH radical was calculated using the following equation: (%) = [1 − (A1 − A0)] × 100 where A1 is the absorbance of the sample and A0 is the absorbance of the blank reaction.

For the spectrophotometric determination of all nonenzymatic antioxidants, an ELISA Micro Plate Reader (LKB 5060–006, Winooski, VT, USA) was used.

### 2.5. Estimation of Enzymatic Antioxidants

Centaury shoots were homogenized in potassium phosphate extraction buffer containing insoluble polyvinylpolypyrrolidone, dithiothreitol and phenyl methyl sulfonyl fluoride. The homogenate was centrifuged at 4 °C for 5 min and the protein content was determined from the supernatant according to Bradford [49] using bovine serum albumin as the standard. The quantification of SOD, CAT and POX was also performed.

SOD activity was determined spectrophotometrically using a modified method from Beyer and Fridowich [50]. The reaction mixture contained potassium phosphate buffer, ethylenediaminetetraacetic acid, methionine, nitroblue tetrazolium chloride (NBT) and riboflavin. The reaction mixtures were added to the samples, which were then illuminated for 1–2 min and the absorbance was measured at 540 nm. One unit of SOD activity is the amount of sample required for 50% inhibition of NBT photoreduction and is presented as the specific activity (U/mg). SOD activity was spectrophotometrically detected using an ELISA Micro Plate Reader (LKB 5060–006, Winooski, VT, USA).

CAT activity was determined spectrophotometrically using the method from Aebi [51]. This method is based on monitoring the kinetics of the consumption of H_2_O_2_, which can be detected by measuring the absorbance (at 240 nm) of the reaction mixture consisting potassium phosphate buffer, H_2_O_2_ and enzyme extract. One unit of CAT activity is defined as the amount of enzyme required to degrade 1 µM of H_2_O_2_ in 1 min and is indicated as µM min^−1^ mg protein^−1^ (U/mg protein).

POX activity was determined spectrophotometrically using the method from Kukavica and Veljović-Jovanović [52]. The reaction mixture contained potassium phosphate buffer and pyrogallol as the enzyme substrate. The POX-catalyzed oxidation of pyrogallol to purpurogallin in the presence of H_2_O_2_ was monitored by absorbance determination at 430 nm. Enzyme activity is indicated as µM min^−1^ mg protein^−1^ (U/mg protein). The absorbances of the CAT and POX reactions were measured with a UV–visible spectrophotometer (Agilent 8453, Life Sciences, USA).

### 2.6. Statistical Analysis

The effect of different SNP pretreatments/treatments on the biochemical parameters of centaury shoots, after four weeks of culture, were evaluated using standard two-factor analysis of variance (ANOVA). All analysed parameters were measured using three biological samples per treatment. In addition, the absorbances of all supernatants were measured in triplicate for each sample. The results are presented as mean ± SE. The comparisons between the mean values were made using a Fisher LSD (the least significant difference) post-hoc test, calculated at a confidence level of *p* ≤ 0.05.

## 3. Results

### 3.1. The Effect of SNP on Photosynthetic Pigments Content during Salt Stress in C. erythreae Shoots

**Figure 2:**
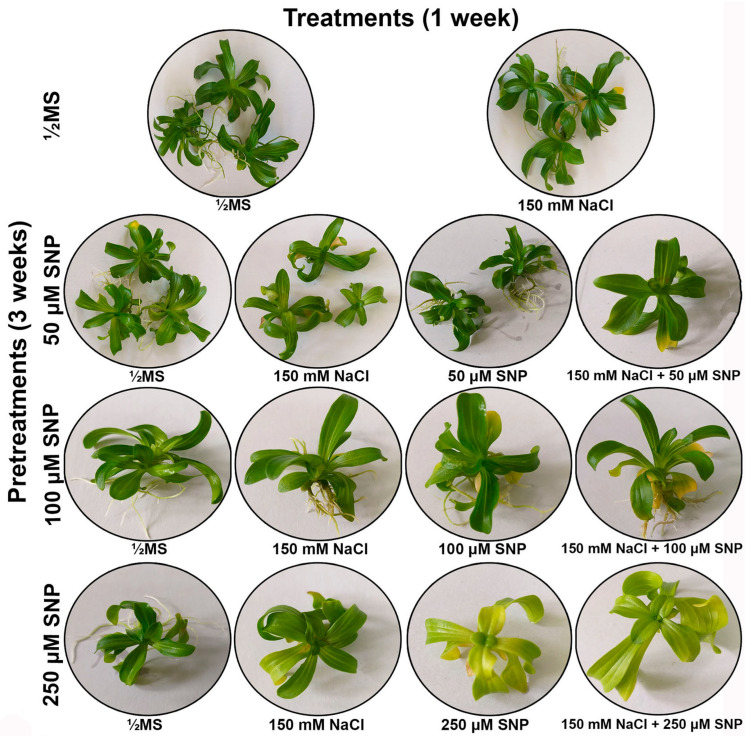
*Centaurium erythraea* shoots after four weeks of cultivation on different SNP pretreatments, NaCl and/or SNP treatments.

The centaury shoots successfully survived four weeks on ½MS media supplemented with different combinations of SNP (0, 50, 100 or 250 μM) and/or NaCl (150 mM). Control centaury shoots grown on NaCl-free medium developed the usual rosette morphology and dark green oval leaves (Figure 2). Pretreatments with 50 and 100 μM SNP altered the color of the leaves to light green. Pretreatment with 250 μM SNP caused leaf tip curling and desiccation as well as yellowing of the leaves and chlorosis of the entire shoot. After this pretreatment, and especially in combination with SNP, and NaCl, the highest number of yellow leaves was observed. Unlike other pretreatments/treatments, only after the pretreatment with 250 μM SNP, most centaury shoots did not spontaneously develop roots.

**Figure 3:**
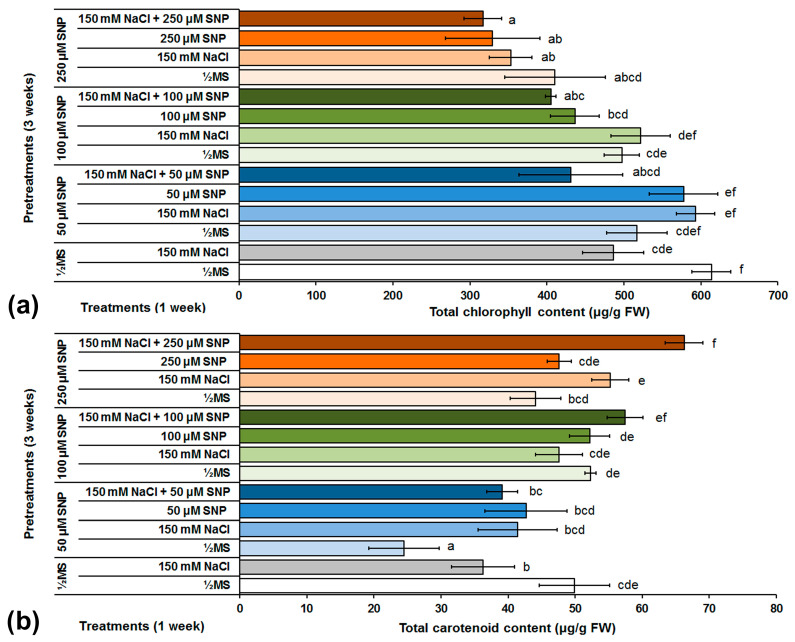
The effect of different SNP pretreatments and NaCl and/or SNP treatments on total chlorophyll (**a**) and carotenoid (**b**) content in *C. erythraea* shoots. Data represent mean ± standard error. Bars marked with a different letter are significantly different from the control according to the LSD test (*p* ≤ 0.05).

Leaf chlorosis is one of the most common symptoms of stress caused by NaCl due to decreased photosynthetic pigments and is also an important indicator of the physiological state of the plants. Therefore, the content of photosynthetic pigments was determined in two control groups of shoots that were grown on ½MS NaCl-free medium throughout the whole experimental period, then in medium supplemented with 150 mM NaCl, as well as in shoots grown on different SNP pretreatments and NaCl and/or SNP treatments. In the second control group of centaury shoots not exposed to SNP during the pretreatment, NaCl decreased total *Chl* content ~21% in comparison to the first control group grown on ½MS NaCl-free medium (Figure 3a). In addition, pretreatment with 50 μM SNP significantly decreased the total *Chl* in shoots grown on NaCl-free medium in comparison to the control group of shoots grown on the same medium. Conversely, the combination of 50 μM SNP pretreatment and then treatments with NaCl and 50 μM SNP, increased total *Chl* content ~20% in comparison to the control group of shoots grown on NaCl-supplemented medium, as well as in comparison to the control shoots from the appropriate treatment. Pretreatment with 50 μM SNP in combination with treatment including NaCl and 50 μM SNP together, also reduced total *Chl* content to the lowest level in this experimental group. The application of 100 μM SNP in the pretreatment, did not lead to significant changes in total *Chl* content in comparison to the control group centaury shoots grown on medium with NaCl. The 250 μM SNP pretreatment did not show any positive effects, and decreased total *Chl* content in comparison to the control group of shoots grown on medium with NaCl. It was interesting to note that the lowest total *Chl* content was detected in centaury shoots exposed to treatments including both NaCl and SNP after the appropriate SNP pretreatments. It was also found that increased SNP concentrations in the pretreatment were negatively correlated with decreased total *Chl* content after the corresponding SNP and NaCl treatments.

The effect of the different SNP pretreatments and NaCl and/or SNP treatments on total carotenoid content is shown in Figure 3b. In the control groups of century shoots, NaCl decreased the total carotenoid content 27% in comparison to the control group of shoots grown on NaCl-free medium. Pretreatment with 50 μM SNP halved the total carotenoid content in shoots grown on NaCl-free medium in comparison to the control group of shoots grown on the same medium. Conversely, pretreatments with 150 and 250 μM SNP did not significantly change the total carotenoid content in comparison to the control group of shoots grown on NaCl-free medium. In shoots grown on medium supplemented with NaCl, pretreatments with 100 and 250 μM SNP increased the total carotenoid content 31 and 52%, respectively, in comparison to control group of shoots grown on the same medium. In addition, in comparison to the control group, the application of 100 and 250 μM SNP as pretreatments in combination with the same SNP concentrations in the treatments, influenced a significant increase in total carotenoid content. Furthermore, pretreatments with 100 and 250 μM SNP, followed by treatments with the same SNP concentrations and NaCl together, resulted in a significant increase in total carotenoid content in comparison to the control group of shoots grown on medium supplemented with NaCl.

### 3.2. The Effect of SNP on Oxidative Stress Biomarkers during Salt Stress in C. erythreae Shoots

**Figure 4:**
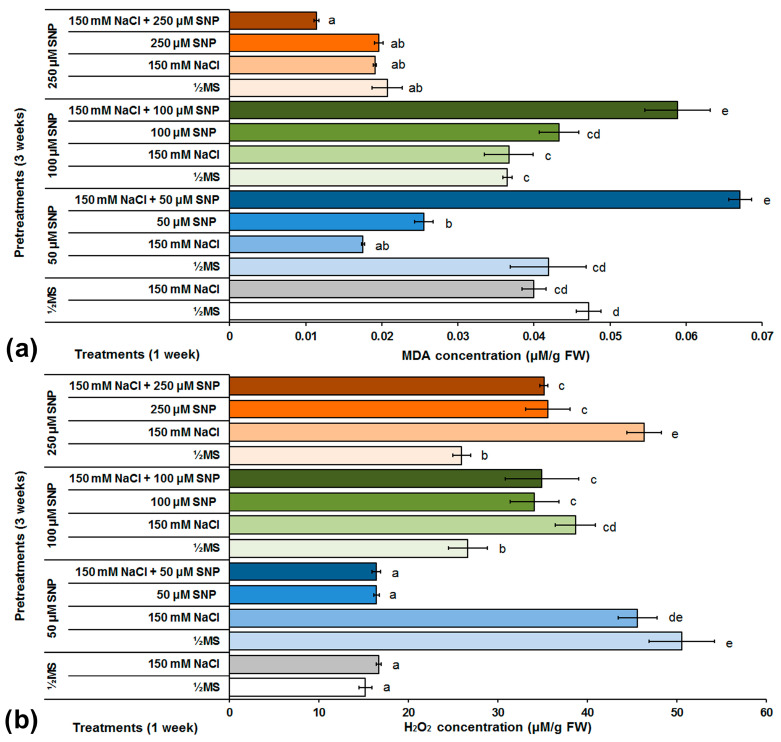
The effect of different SNP pretreatments and NaCl and/or SNP treatments on MDA (**a**) and H_2_O_2_ (**b**) concentrations in *C. erythraea* shoots. Data represent mean ± standard error. Bars marked with a different letter are significantly different from the control according to the LSD test (*p* ≤ 0.05).

The effect of different SNP pretreatments and NaCl and/or SNP treatments on level of lipid peroxidation in centaury shoots was determined by monitoring the MDA concentration (Figure 4a). In the control group cultured on medium supplemented with NaCl, a decrease in MDA concentration (15%) was observed in comparison to the control group grown on NaCl-free medium. All SNP pretreatments significantly reduced MDA concentrations in the centaury shoots grown on NaCl-supplemented medium, especially the 50 and 250 μM SNP pretreatments, where the MDA concentrations were reduced to 56 and 52%, respectively, in comparison to the control group grown on NaCl. Treatments with 50 and 250 μM SNP decreased MDA concentration, while 100 μM SNP did not significantly change the MDA concentration in comparison to both control groups. A significant increase in lipid peroxidation was observed after treatments with a combination of 50 or 100 μM SNP with 150 mM NaCl. In addition, the highest degree of lipid peroxidation, compared to all treatments tested, was detected after the treatment using 250 μM SNP and 150 mM NaCl.

Since lipid peroxidation is one of the consequences of oxidative stress, H_2_O_2_ concentration was also determined as a marker of the degree of plant cell oxidative damage (Figure 4b). The two control groups had approximately the same H_2_O_2_ concentrations. Pretreatments with 50, 100 or 250 SNP concentrations increased H_2_O_2_ in shoots grown on NaCl-free medium by about 233, 75 and 71%, respectively, in comparison to the control centaury shoots grown on NaCl-free medium and by about 173, 131 and 177%, respectively, in comparison to control shoots grown on NaCl medium. Treatment with 50 μM SNP did not significantly change the H_2_O_2_ concentration in comparison to both control groups. Conversely, treatments with 100 and 250 μM SNP significantly increased H_2_O_2_ concentration in comparison to both control groups. The same pattern was also observed in all SNP treatments in combination with NaCl.

### 3.3. The Effect of SNP on Nonenzymatic Antioxidants during Salt Stress in C. erythreae Shoots

**Figure 5:**
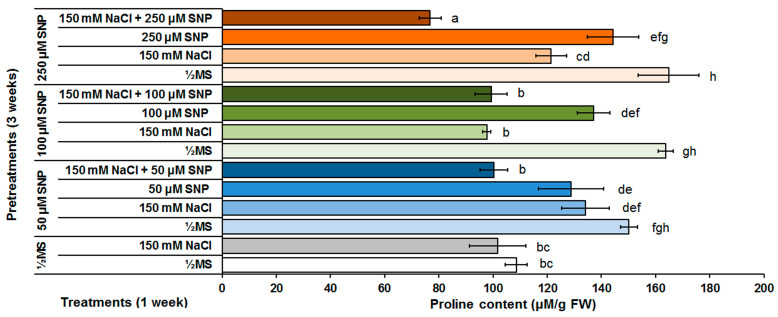
The effect of different SNP pretreatments and NaCl and/or SNP treatments on proline content in *C. erythraea* shoots. Data represent mean ± standard error. Bars marked with a different letter are significantly different from the control according to the LSD test (*p* ≤ 0.05).

The centaury control shoots grown under unstressed and NaCl-stressed conditions in vitro has similar free proline contents (Figure 5). After pretreatment with 50, 100 or 250 μM SNP, increased proline content (38, 50 and 52%, respectively) was observed in shoots grown on ½MS nutrient medium in comparison to the control group of shoots grown on the same medium. Only pretreatment with 50 μM SNP resulted in a significant increase in proline content (32%) after NaCl treatment in comparison to the control group of centaury shoots grown on medium supplemented with NaCl. Increased SNP concentrations, using the same concentration in pretreatments and in following treatments, was positively correlated with increased proline content in comparison to both control groups. However, treatments with all SNP concentrations showed lower levels of proline content in comparison to the corresponding treatments control. On the other hand, pretreatments with 50 and 100 μM SNP followed by treatments with the same SNP concentrations and NaCl together, decreased proline content to the control values of stressed shoots, while the lowest proline content, lower than in both control groups, was detected in centaury shoots grown on treatment with 250 μM SNP and NaCl together.

**Figure 6:**
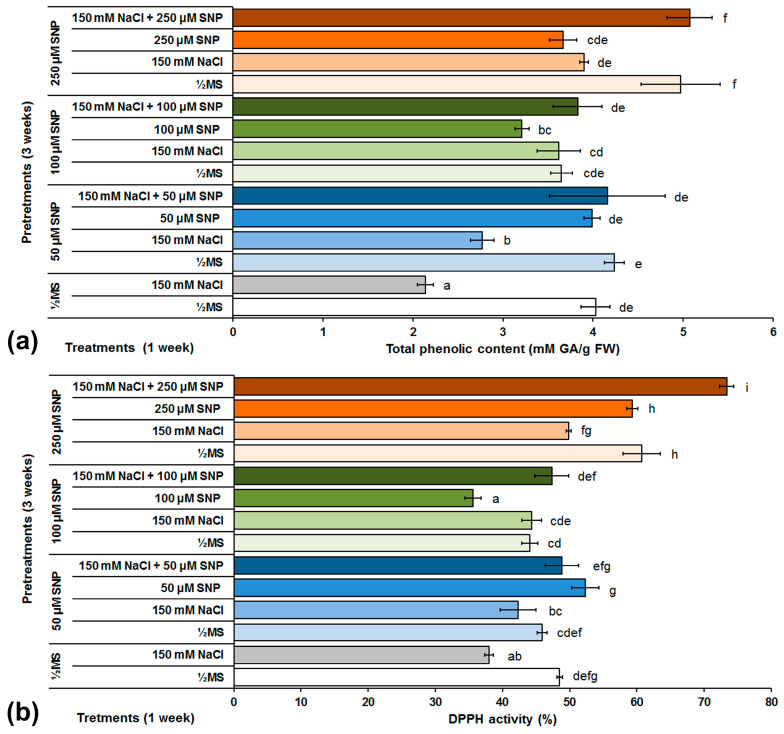
The effect of different SNP pretreatments and NaCl and/or SNP treatments on total phenolic content (**a**) and DPPH concentration (**b**) in *C. erythraea* shoots. Data represent mean ± standard error. Bars marked with a different letter are significantly different from the control according to the LSD test (*p* ≤ 0.05).

The amount of total phenolic compounds in centaury shoots exposed to different SNP pretreatments and/or treatments was determined (Figure 6a). In the control centaury shoots grown on NaCl-free medium, similar total phenolic content was detected, in comparison to shoots grown on NaCl-supplemented medium. Pretreatments with 50 and 100 μM SNP in NaCl-free medium did not significantly change the amount of total phenolic content in comparison to the corresponding control group, while pretreatment with 250 μM SNP increased the amount of total polyphenols by about 23%. Conversely, all applied SNP pretreatments (50, 100 and 250 μM) caused significant increase in the total phenolic content (29, 69 and 82%, respectively) in shoots grown on medium supplemented with NaCl in comparison to control shoots grown on the same medium. In addition, the application of all SNP concentrations in the pretreatments and treatments, increased the total phenol content in comparison to control shoots grown on NaCl, but these levels still did not exceed the values recorded in control shoots grown on NaCl-free medium. The same pattern was observed after all treatments that included the combinations of 50 or 100 μM SNP and NaCl. The only exception was the combination of 250 μM SNP and NaCl, where an increase of about 26% was observed in comparison to control shoots grown on ½MS medium.

The influence of the different SNP pretreatments and NaCl and/or SNP treatments on the antioxidant capacity of centaury shoots is presented on Figure 6b. In control conditions, the addition of NaCl decreased the DPPH concentration by 28% in comparison to shoots grown on ½MS medium. In comparison to control shoots grown on NaCl-free medium, pretreatments with 50 and 100 μM SNP did not significantly change DPPH concentrations while pretreatment with 250 μM SNP significantly increased DPPH in shoots grown on the same medium. Under the conditions of salt stress caused by NaCl, pretreatments with all SNP concentrations (50, 100 and 250 μM) shown an increase in the degree of DPPH reduction by 11, 17 and 31%, respectively, in comparison to the corresponding control group. Treatments with 50 and 100 μM SNP did not significantly alter DPPH concentrations and both values were similar to control shoots grown on ½MS and NaCl-free medium, respectively. Only treatment with 250 μM SNP, significantly increased DPPH concentration in comparison to both control groups, but still at the level of control shoots within the same treatment. Using the combination treatments containing NaCl and 50 or 100 μM SNP, an increased DPPH was detected in comparison to control shoots grown on NaCl, but DPPH concentration was not changed in comparison to the second group of shoots grown on ½MS medium. Among all treatments tested, the most significant degree of DPPH reduction, in comparison to both control groups, was recorded in shoots grown on media supplemented with NaCl and 250 μM SNP.

### 3.4. The Effect of SNP on Enzymatic Antioxidants during Salt Stress in C. erythreae Shoots

**Figure 7:**
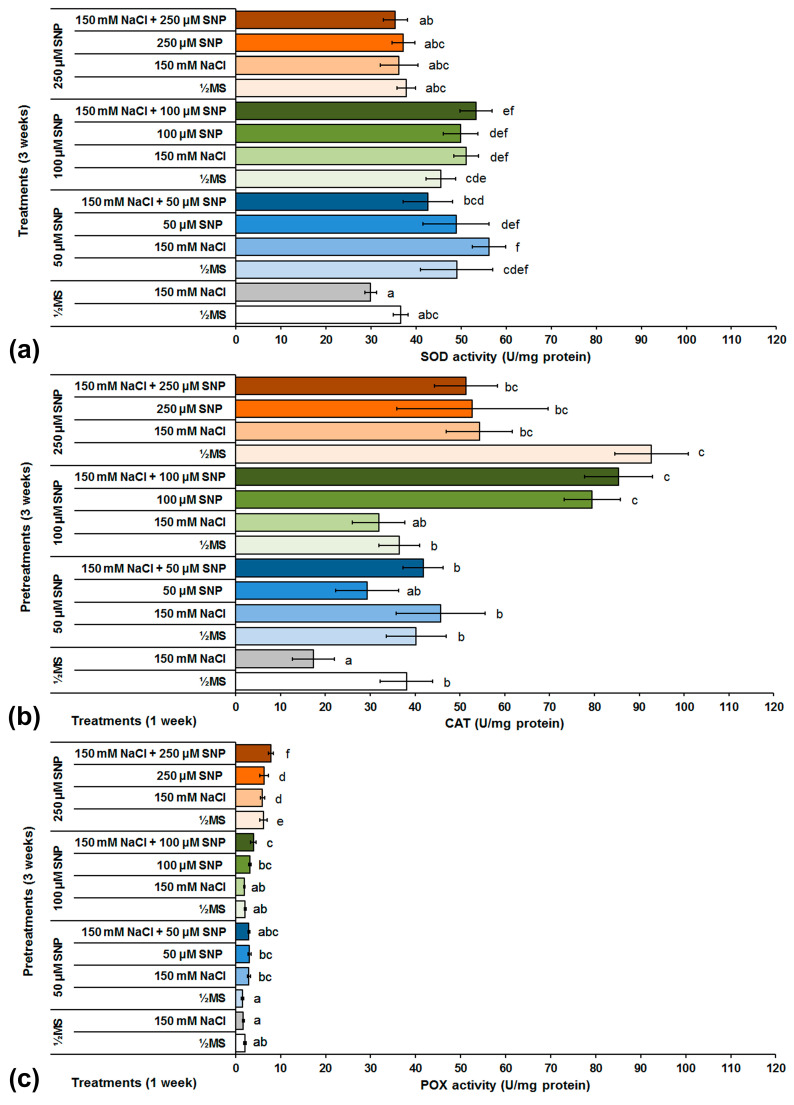
The effect of different SNP pretreatments and NaCl and/or SNP treatments on SOD (**a**), CAT (**b**) and POX (**c**) activities in *C. erythraea* shoots. Data represent mean ± standard error. Bars marked with a different letter are significantly different from the control according to the LSD test (*p* ≤ 0.05).

In the control groups of shoots grown in the presence of NaCl, SOD activity was decreased by about 18% in comparison to control shoots grown on NaCl-free medium (Figure 7a). In shoots grown on ½MS medium, the 50 and 100 μM SNP pretreatment increased SOD activity by about 34 and 24%, respectively, while pretreatment with 250 μM SNP did not significantly changed SOD activity in comparison to control shoots grown on the same medium. In shoots grown on medium supplemented with NaCl and previously pretreated with 50, 100 and 250 μM SNP, the same pattern was observed. SOD activity was increased by 88 and 71% after the application of 50 and 100 μM SNP, respectively, while after 250 μM SNP treatment, SOD activity was similar to control shoots. The application of the SNP treatments caused an increase in SOD activity in comparison to both control groups. However, it was interesting to note that the increasing SNP concentrations were inversely correlated with increasing SOD activity. The highest SOD activity among all the treatment combinations was recorded in shoots grown on NaCl and 100 μM SNP. By increasing the SNP concentration to 250 μM along with the NaCl treatment, the SOD activity decreased to the control level of the corresponding treatment and the control shoots grown on ½MS or medium supplemented with NaCl.

Similar to the SOD activity, in control conditions, CAT activity was also decreased (by about 55%) in shoots grown on NaCl medium (Figure 7b). Pretreatment with 50 μM SNP did not significantly change CAT activity in comparison to both control groups. Treatment with 100 μM SNP, individually or together with NaCl, significantly increased CAT activity. In the shoots grown on NaCl-free medium, a significant increase in CAT activity was recorded only after 250 μM SNP pretreatment. At the same time, this is the highest recorded CAT activity in the centaury shoots after all applied treatments, and represents an increase of 143% in comparison to control shoots grown on ½MS medium.

Similar to SOD and CAT activities in the control groups, POX activity was also decreased (by approximately 17%) in shoots grown on NaCl medium (Figure 7c). No significant changes in POX activity were observed in shoots pretreated with 50 or 100 μM SNP and grown on both MS and NaCl-free media. Pretreatments with 50 or 100 μM SNP and furtherr culture on media with the same SNP concentrations and NaCl together, increased POX activity). A significant increase in POX activity was observed after pretreatment with 250 μM SNP and all further treatments. Thus, POX activity was tripled in centaury shoots pretreated with 250 μM SNP and further grown on NaCl-free medium, in comparison to control shoots grown on the same medium. The same pattern was also observed in shoots grown on NaCl and control shoots grown on the same medium but not treated with SNP. Similar POX activity changes were detected in shoots treated only with 250 μM SNP. In comparison to all applied treatments, the highest POX activity was recorded in centaury shoots grown on medium supplemented with 250 μM SNP and NaCl together.

## 4. Discussion

Although in nature, centaury inhabits mountain slopes, dry grasslands, scrublands and saline soils, investigations of centaury’s response to stressful conditions in vitro are still at the beginning stages. The role of the widely used NO donor, SNP, on plant tolerance to salt stress conditions is usually demonstrated after foliar treatment or using nanoparticles [53]. In this work, the effect of exogenously applied SNP, alone or in combination with NaCl, on several biochemical parameters of centaury shoots grown in vitro was investigated.

### 4.1. SNP and Photosynthetic Pigments during Salt Stress in C. erythreae

Due the importance of photosynthesis, as a key physiological process in plants, the effect of different SNP pretreatments and NaCl and/or SNP treatments on the concentration of photosynthetic pigments of centaury was determined. This work demonstrated that total *Chl* content was significantly decreased in control shoots grown on NaCl in comparison to the other control group of shoots grown on ½MS medium (Figure 3a). These results are in accordance with the results previously obtained in centaury shoots grown during NaCl-caused salt stress in vitro [37,39]. The lowest SNP concentration applied at pretreatment (50 μM) shown a positive effect on total *Chl* content in centaury leaves during salt stress. Conversely, the highest SNP concentration (250 μM) decreased total *Chl* content to levels lower than the control group of shoots grown on NaCl. These results could be expected because in addition to oxidative stress, centaury shoots were also exposed to higher intensity of nitrosative stress. The positive effect of SNP on total *Chl* content under stress conditions caused by NaCl was also confirmed in cotton, red raspberry, barley, sunflower and wheat [25,28,54,55,56]. It was interesting to note that SNP pretreatments did not increase total *Chl* content in comparison to the control group of shoots grown on NaCl-free medium. However, some reports showed that SNP treatment increased total *Chl* content in cotton and raspberry plants grown under salt stress in comparison to control conditions [25,54]. In summary, it can be assumed that a lower SNP concentration had a positive effect on *Chl* preservation by promoting the synthesis, regeneration and/or inhibiting its degradation but also promoting the mechanisms that remove ROS, and the ability of SNP to improve the K^+^/Na^+^ ratio [25,27].

The results presented in this work showed that NaCl had negative effect on total carotenoid content in centaury shoots grown in control conditions (Figure 3b). Decreased carotenoid content was also recently reported in centaury shoots under salt stress in vitro [39]. In centaury shoots treated with NaCl, the application of SNP pretreatments resulted in increased total carotenoid content in comparison to control group of shoots grown on medium also supplemented with NaCl. The highest total carotenoid content was observed after treatment with 250 μM SNP and NaCl together. These findings, describing the positive effect of SNP on carotenoid content, correspond with published results from cotton, red raspberry and sunflower plants [25,54,56]. It is quite possible that carotenoids, as non-enzymatic antioxidants, prevent or minimize the oxidative damage induced by NaCl. The latest research proposed increased carotenoid content as a marker of salt tolerance [57]. Accordingly, it can be concluded that SNP increased centaury’s tolerance to salt stress.

### 4.2. SNP and Oxidative Stress Biomarkers during Salt Stress in C. erythreae

In control conditions, a decrease in MDA content was observed in centaury shoots during salt stress in comparison to shoots grown on NaCl-free medium (Figure 4a). This result is unexpected because, theoretically, exposure to salt stress should increase the degree of lipid peroxidation. It is possible that the duration and/or level of stress intensity were not sufficient. However, similar results were also recorded in the halophyte species *Prosopis strombulifera* and *Salvadora persica*, as well as in soybean and a salt-tolerant cultivar of date palm, where no significant changes in MDA content under NaCl-induced stress was detected [58,59,60,61]. Pretreatments with 50 and 250 μM SNP decreased MDA content in NaCl-treated centaury shoots in comparison to both control groups. The effect of SNP on the reduction of MDA content was also shown in other plant species such as cotton, wheat, apple and lentil [25,27,28,29]. The most interesting results, in terms of lipid peroxidation, were obtained in centaury shoots treated with SNP and NaCl together. The highest MDA content was obtained with the application of 50 μM SNP and NaCl whereas the lowest recorded rate of lipid peroxidation was obtained with the application of 250 μM SNP and NaCl. According to certain studies, SNP application can reduce the activity of lipoxygenases and thereby reduce the degree of lipid peroxidation. In addition, NO has the ability to remove peroxyl radical and prevent further oxidative damage [62,63]. However, at low concentrations, NO, together with O_2_^•-^, forms peroxynitrite, which has the ability to initiate lipid peroxidation [17,53].

In control conditions, salt stress caused a slight increase in H_2_O_2_ content in comparison to shoots grown on NaCl-free medium (Figure 4b). On the same media, pretreatments with all SNP concentrations induced significant H_2_O_2_ production in centaury shoots, with higher H_2_O_2_ content after salt stress. This result can be explained by considering H_2_O_2_ not only as oxidative stress marker, but also as a signaling molecule that is important for the establishment of salinity tolerance [64,65]. The application of all SNP concentrations, alone or in combination with NaCl, reduced H_2_O_2_ content in centaury shoots after all tested treatments. This reduction may be responsible for the induction of antioxidant defense system to scavenge H_2_O_2_. These results are in accordance with SNP application reducing the H_2_O_2_ content in cucumber, lettuce, wheat, brown mustard and lentil [23,28,29,66,67].

### 4.3. SNP and Nonenzymatic Antioxidants during Salt Stress in C. erythreae

The accumulation of endogenous proline content under salinity conditions can be considered as a marker of plant stress tolerance [14]. Increased proline content during exposure to NaCl-induced stress has been documented in numerous plant species including centaury [38]. The results obtained during this investigation showed that the application of all SNP pretreatments increased the proline content in centaury shoots grown on NaCl, similar to those grown on ½MS medium, in comparison to both control groups (Figure 5). In addition, it was noted that all SNP concentrations in treatments were positively correlated with increased proline content. It is obvious that SNP alone, as a potential stressogenic factor, further induced proline accumulation in centaury shoots, likely with enhanced activity of proline-synthesizing enzymes, together with a reduction in proline catabolism under stress conditions [68]. On the contrary, pretreatments including combinations of SNP and NaCl together, reduced free proline content in centaury shoots. Many studies indicated that NO is involved in proline metabolism during stress conditions but the detected effects were different. Some reports revealed increased proline content in SNP-treated *Lactuca sativa* [66], *Pisum sativum* [69] and *Brassica chinensis* [70] under saline stress. Conversely, reduced proline content, as a consequence of SNP pretreatment, was detected in cucumber [23] and *Brassica rapa* [71] under salt stress. All of these results imply that enhanced proline content is not always essential for plant stress tolerance response because the accumulation of this osmolyte does not always correlate with better plant responses, as in case of NaCl-treated centaury shoots. In addition, considering that synthesis of different osmolytes is an “energetically expensive” process, it is possible that centaury activates other mechanisms with lower energy demands, for example, efficient ions compartmentalization to achieve salinity tolerance [72].

Phenolic compounds belong to the group of secondary metabolites that participate in numerous physiological processes in plants; one of those roles is ROS scavenging under various environmental stresses [73]. Although in most plant species total phenolic content increased under high salinity, there are reports describing decreased phenol content in *Phaseolus vulgaris* and *Schizonepeta tenuifolia* grown under salt stress conditions [74,75]. The same result was observed in NaCl-treated centaury shoots (Figure 6a). The effect of SNP on the total phenolic content increase under NaCl stress conditions was previously documented in mangrove species *Aegiceras corniculatum*, wheat, sunflower, and apple [27,56,76,77]. A similar result was detected in centaury shoots pretreated with SNP and then grown on ½MS medium or medium supplemented with NaCl. Treatments with all SNP concentrations also increased total phenolic content, while the highest increment among all the treatments was recorded in centaury shoots after combination treatments with all SNP concentrations and NaCl together. During abiotic stress, NO can increase the activity of phenylalanine ammonia-lyase (PAL) and consequently enhance phenolic compounds biosynthesis [17]. The increased activity of the PAL enzyme could be the reason for the increased total phenolic content in centaury shoots after exposure to SNP.

Due to its ability to react with antioxidants, the DPPH radical is a good indicator of the antioxidant capacity of plants [78]. The results obtained in this work showed that, in control conditions, centaury shoots grown on a medium supplemented with NaCl had decreased antioxidant capacity in comparison to shoots grown on a NaCl-free medium (Figure 6b). This result is in accordance with the previous reports where decreased DPPH concentration under NaCl-induced stress in cucumber, sage, spinach, henbane and flax was described [23,79,80,81,82]. In order to investigate the changes in centaury antioxidant capacity, the influence of SNP pretreatments on DPPH concentration was tested. The results showed that, in general, all SNP pretreatments increased DPPH concentration in centaury shoots. The largest DPPH concentration was detected in shoots grown on a combination medium supplemented with SNP and NaCl together. These changes in DPPH concentrations, based on their free radical scavenging capacities, positively correlated with total phenolic content in centaury shoots. Furthermore, in several medicinal herbs and selected species of wild vegetables, total phenolic amounts were also significantly correlated with antioxidant capacity [83,84].

### 4.4. SNP and Enzymatic Antioxidants during Salt Stress in C. erythreae

Various stress conditions can induce ROS production, which leads to a change in enzyme activity in order to maintain homeostasis in plant cells. Antioxidant enzymes that play a significant role in removing ROS forms and protecting plant cell structures from oxidative stress, include SOD, CAT and POX [16]. Increased SOD, CAT and POX activities under NaCl stress have been documented in many species including sunflower and oilseed rape [56,64]. In this work, decreased activities of SOD, CAT and POX were observed in centaury shoots grown under stress conditions caused by NaCl. Although unexpected, the same results were also reported in halophytic species *Salvadora persica*, date palm and the oil-seed crop *Brassica juncea* [60,61,67]. The positive effect of SNP on the activity of SOD, CAT and POX was previously confirmed in citrus seedlings, wheat and lentil under salinity stress [28,29,85]. The application of SNP increased SOD activity in centaury shoots grown under NaCl, as well as in shoots grown on NaCl-free medium in comparison to the corresponding control groups (Figure 7a). The highest SOD activity was recorded after 50 μM SNP pretreatment while increased SNP concentration decreased SOD activity in centaury shoots. The same trend was also observed in cotton seedlings [25]. The application of SNP pretreatments also increased CAT activity in centaury shoots grown under NaCl-induced stress conditions as well as in shoots grown on nutrient media without NaCl (Figure 7b). The highest CAT activity was determined after the application of 250 μM SNP pretreatment. It can be concluded that SNP stimulated CAT activity in centaury shoots, which has also been observed in tomato and sunflower [22,56]. As in the case of CAT, the same pattern in POX activity was observed. SNP pretreatments increased POX activity, with the highest activity recorded after 250 μM SNP pretreatment (Figure 7c). Similar results were recorded in cotton and sunflower plants grown under salinity stress conditions [25,56]. It is known that the addition of signaling molecules such as NO and hydrogen sulfide (H_2_S), stimulates the activity of antioxidant enzymes [86]. The role of NO in salt tolerance has been studied in numerous plant species, and there is evidence that the application of NO donors protects plants from salt stress by increasing antioxidant enzyme activity [21]. All the results suggest that NO mitigates the salt-induced oxidative stress by enhancing the activity of enzymatic antioxidants, thus improving centaury’s tolerance to salt stress caused by NaCl.

## 5. Conclusions

**Figure 8:**
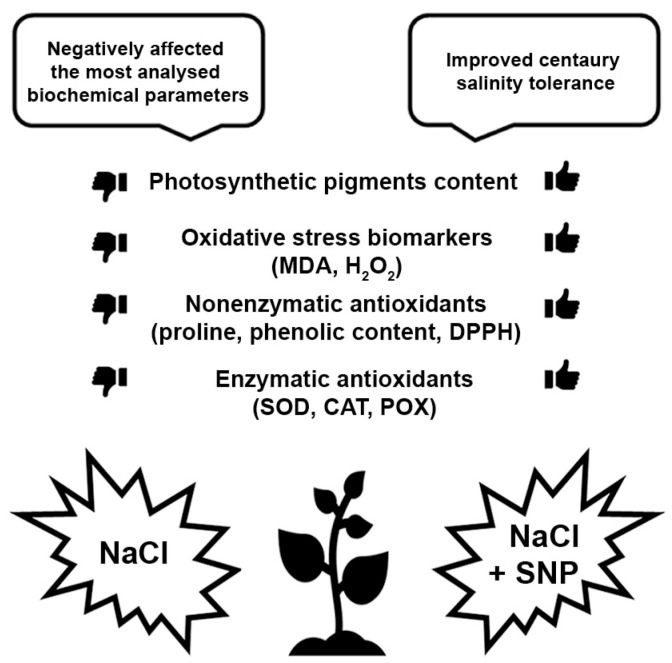
Schematic illustration showing how SNP affects centaury shoots during salt stress in vitro.

Centaury shoots grown under NaCl-induced stress decreased the content of photosynthetic pigments, total phenolic compounds and DPPH. The activities of SOD, CAT and POX were also reduced under salt stress conditions. All these results indicate that centaury is a salinity-sensitive plant species. However, the MDA concentration was decreased while H_2_O_2_ concentration did not drastically change under stress conditions caused by NaCl, which indicate that centaury can be also be considered a salinity-tolerant species. Under salt stress conditions, proline content also did not significantly change which is not an attribute of salinity-tolerant species. In addition, it is possible that centaury has a preference for other osmolytes, rather than proline. In salt stress conditions, Na^+^ and Cl^-^ ions can act as “cheap osmolytes”. In addition, the effective removal of Na^+^ from the cytosol does not result in excessive ROS generation, eliminating the high activity of antioxidant mechanisms. Therefore, it is necessary to investigate the mechanisms that regulate the transport of ions in centaury in order to reveal if this important medicinal plant is a halophytic species. The results presented in this work also shown that SNP, a widely used NO donor, improved centaury tolerance to salinity (Figure 8). SNP showed a positive effect on total *Chl* and carotenoid content and affected lipid peroxidation, proline and total phenolic content, DPPH concentrations as well as antioxidant enzyme activities in centaury shoots grown under salt stress caused by NaCl. In addition to NO, SNP releases cyanide and iron ions as toxic by-products, and thus limits its potential application in agriculture. Therefore, nanoparticles that release NO, as well as S-nitrosothiols and S-nitrosoglutathione, the natural reservoirs of NO in biological systems, have been suggested as alternatives to SNP application.

## Data Availability

All the data are contained within the manuscript.
